# Neurocognitive difficulties in trauma-exposed adults with metabolic syndrome: no influence of PTSD status or PTSD and metabolic syndrome comorbidity

**DOI:** 10.1007/s44192-025-00141-5

**Published:** 2025-02-20

**Authors:** Sharain Suliman, Leigh van den Heuvel, Erine Bröcker, Soraya Seedat

**Affiliations:** 1 Department of Psychiatry, Faculty of Medicine and Health Sciences, Stellenbosch University, Cape Town, 7550 South Africa; 2 South African Medical Research Council Genomics of Brain Disorders Research Unit, Department of Psychiatry, Faculty of Medicine and Health Sciences, Stellenbosch University, Stellenbosch University, Cape Town, 7550 South Africa

**Keywords:** Adults, Metabolic Syndrome; Neurocognition; Posttraumatic Stress Disorder; Trauma-exposed

## Abstract

**Background:**

Metabolic syndrome (MetS) and posttraumatic stress disorder (PTSD) often co-occur and both may compromise cognition, owing in part to common underlying mechanisms. Few studies have investigated the additive effects of these disorders on cognitive performance. Our aims were to compare cognitive performance between patients with PTSD and trauma-exposed controls (TEC) and investigate the additive effects of MetS factors on cognition.

**Methods:**

In this case–control study, we included 474 adult participants, 236 with PTSD and 238 TEC. Demographic, neuropsychiatric, metabolic-related, and neurocognitive assessments were undertaken and MANCOVAs performed controlling for age. Cognitive domains (immediate and delayed memory, attention, language, visuospatial performance, working memory and global cognition) were the dependent variables in the analysis. Patient status and presence/absence of MetS or MetS components were independent variables, in each model.

**Results:**

Patients with PTSD did not demonstrate worse cognitive performance than TEC on the neurocognitive domains assessed, and the presence of MetS in patients with PTSD did not alter this finding. Individuals with MetS also did not demonstrate worse cognition when compared to those without MetS. When we looked at individual MetS features, higher BMI was associated with poorer visuospatial performance,

**Conclusions:**

These findings contrast with many previous studies showing worse neurocognitive performance related to both PTSD and MetS. Further investigation is required to establish the contribution of MetS to cognitive deficits in those with PTSD. Generalisability and inferences regarding the directionality of associations are limited.

## Introduction

Metabolic syndrome (MetS) refers to a cluster of metabolic abnormalities that frequently coexist and are risk factors for cardiovascular disease [1]. These risk factors include elevated waist circumference, raised triglycerides (TG), low high-density lipoprotein cholesterol (HDL), raised blood pressure (BP), and raised glucose, and are often associated with obesity [1]. MetS has been associated with a number of psychiatric disorders, including psychotic disorders, bipolar disorder and depression possibly due shared pathophysiological features, such as hypothalamic–pituitary–adrenal and mitochondrial dysfunction, neuro-inflammation, common genetic links and epigenetic interactions [2].

There is a well-established link between posttraumatic stress disorder (PTSD), a neuropsychiatric disorder that can develop after a potentially traumatic event, and MetS. Meta-analytic studies have found that individuals with PTSD have a greater risk of MetS, as well as of the individual MetS features [3–5]. Obesity was found to be elevated in those with PTSD [3, 6]. Some authors have framed PTSD as a risk factor for MetS, while others have hypothesized that the association between PTSD and cardiovascular disease is underpinned by both behavioral and biological mediators, such as accelerated cellular aging through epigenetic effects [7, 8]. A review by Michopoulos and colleagues [10] raised the possibility that PTSD is itself a metabolic disorder and proposed altered inflammatory responses as a common underlying mechanism [9]. Bartoli and colleagues [11] hypothesized that a dysfunctional adaptation to stress might increase the vulnerability to both metabolic dysfunction and PTSD after traumatic experiences [10].

Individuals with NPDs, such as PTSD, frequently demonstrate neurocognitive impairments and subjective complaints of cognitive difficulties. These cognitive difficulties have been implicated in the development and maintenance of PTSD symptoms and contribute to problems in both occupational and social functioning [11–15]. Meta-analyses and qualitative reviews consistently show that when compared to trauma-exposed (TEC) and unexposed controls, individuals with PTSD perform worse on measures of learning, and visual and verbal memory [13, 16–21]. This aligns with cognitive theories of PTSD that suggest inadequate encoding of the trauma memory and poorer memory processes may be associated with greater risk of PTSD symptoms and development of PTSD following trauma exposure [14, 22–24].

Reviews (not all systematic) and a meta-analysis focusing on executive functioning, including attention and sustained attention, provide convergent evidence that individuals with PTSD are impaired in these domains [17, 18, 21, 25–27]. This is in line with models that emphasize the role of cognitive control in the development and maintenance of hyperarousal and re-experiencing symptoms [26]. Poor cognitive control may result in difficulties with management of emotional and behavioral responses in stressful contexts [14]. Additionally, attentional control theory, suggests that hypervigilance might interfere with other neurocognitive processes such as learning and memory by drawing attention towards threatening stimuli [28].

Significant impairments were found for information processing speed and language. Information processing biases in PTSD may lead to a over-processing of trauma-related or threatening cues, at the expense of neutral information [29]. Poorer language/ verbal fluency, in individuals with PTSD, was linked to impairments in fear extinction, contextual encoding and emotional regulation [21, 30–32]. As intrusions are often visual representations of traumatic memories, visuospatial tasks have repeatedly been examined, with poorer performance hypothesised to be related to hippocampal dysfunction, a core feature of PTSD [33, 34]. However, variable findings have been observed for visuospatial and visuoconstruction skills and psychomotor processing speed, and no impairments for basic motor processes.

MetS and its features are known to affect cognition and increase the risk for dementia in the elderly [35, 36]. In their review of studies in younger adult populations, Yates and colleagues [40] reported that MetS and its components have a negative impact on cognition, with specific deficits in memory, visuospatial abilities, executive functioning, processing speed, and overall intellectual functioning [37]. Another review that aimed to systematically evaluate cross-sectional associations between MetS and domain specific cognitive performance concluded that individuals with MetS consistently have poorer performance on non-verbal executive function tasks [38]. Findings with regard to verbal fluency, attention, working memory, information processing, memory, language, construction and motor performance domains were less consistent, and visuo-perceptual skills were not found to be affected [38]. Although the mechanisms that underlie the associations between MetS components and cognitive function are still not well established, several studies have identified an interaction between the metabolic syndrome, inflammation, and increased risk of cognitive impairment [39–42].

Few studies have assessed the effect of the double burden of PTSD and MetS on neurocognition. The addition of MetS to PTSD may compound the deleterious effects of each on cognitive performance. A study in military veterans found those with MetS demonstrated poorer performance on tasks of executive function and immediate verbal memory regardless of PTSD status, but that there was an interaction between MetS and PTSD on delayed verbal memory [43], suggesting that the negative impact of MetS on verbal memory was only significant for veterans without PTSD. The authors suggest the possibility that some of the cognitive difficulties experienced by the veterans with PTSD may be due to the presence of cerebrovascular risk factors [44]. Inflammation may be one mechanism underlying the relationship of both PTSD and MetS to poorer cognition [9]. A neuroimaging study, also in veterans, reported that PTSD predicted MetS, which, in turn, was associated with reduced cortical thickness [45]. As cortical thickness has been associated with cognition, this raises concerns of cognitive decline [46, 47].

To our knowledge, no study has assessed cognition in a community sample of adults with PTSD and MetS in a low- and middle-income country (LMIC) or in an African setting. Additionally, as seen above, few studies have focused on the relationship between PTSD, MetS and cognition. MetS may facilitate the development of cognitive difficulties in patients with PTSD, and the interplay between MetS, PTSD and cognitive difficulties may result in worse outcomes and under-representation of these populations in research. Given this gap in the literature, we aimed to determine, in a case–control study, whether cognition is compromised in a South African adult sample with PTSD and whether MetS further compromises cognition. We therefore aimed to compare neurocognitive performance in: (i) trauma-exposed individuals with and without PTSD; (ii) individuals with and without MetS; and (iii) assess whether there was an interaction between PTSD and MetS on neurocognitive performance. In exploratory analysis, we also investigated the relationships between individual MetS features and neurocognitive performance.

We hypothesized that individuals with either PTSD or MetS/MetS features would have poorer neurocognitive performance; and that the combined effect of PTSD and MetS would be greater than the individual effect of each.

## Methodology

### Procedures

Purposive sampling was utilized to recruit participants: active recruitment within communities by a registered nurse and word of mouth, advertisements in community radio and newspapers, and fliers. Participants who reported one or more traumatic events on screening (using the Life Events Checklist for DSM-5 (LEC-5; [48]) were invited to come in for the assessments.

We included 474 adult participants who took part in a cross-sectional case–control project titled “Understanding the SHARED ROOTS of Neuropsychiatric Disorders and Modifiable Risk Factors for Cardiovascular Disease.” Patients meeting Diagnostic and Statistical Manual of Mental Disorders, Fifth Edition (DSM-5) criteria for PTSD (based on the Clinician-Administered Posttraumatic Stress Disorder Scale for DSM–5; (CAPS-5) [49], were included. Control participants (individuals not meeting PTSD status on the CAPS-5) were group-matched to patients based on sex and MetS status. The study was approved by the Health Research Ethics Committee of the Faculty of Medicine and Health Sciences (FHMS), Stellenbosch University (SU), (N13/08/115), and conducted in accordance with the Declaration of Helsinki, and all participants provided written informed consent.

### Participants

Adult participants (aged 18 years and older), self-identifying as mixed-ancestry (South African Coloured) ethnicity were included. Participants were excluded if they were unable to read, write or understand the informed consent documents, diagnosed with a current major medical condition (e.g., epilepsy, cancer, HIV, stroke) or other major psychiatric disorder (e.g. schizophrenia, other psychotic disorder, bipolar disorder), had experienced a significant head injury with loss of consciousness and/or posttraumatic amnesia and, or were pregnant. In addition, control participants with any current psychiatric disorder including substance use disorder, common mental disorder (e.g., anxiety or depressive disorders), or on chronic psychiatric medication at the time were excluded. Since only a small number (n = 21) of PTSD control participants were trauma unexposed we excluded them. Participants who did not complete the neurocognitive assessment were also excluded. This left us with 474 participants (PTSD patients: 236, TEC: 238).

### Measures

1. Demographic Questionnaire: Demographic variables included age, sex, highest level of education obtained (HLOE), employment status, income and alcohol and drug usage in the previous six months.
2. Metabolic Syndrome: MetS diagnosis was based on harmonized JIS criteria, which includes the presence of three or more of the following [1]: raised waist circumference (WC; ≥ 90 cm for both men and women as a validation study in the Western Cape showed this to be the optimal cut-off in mixed-ancestry men and women) [50]; raised triglycerides (TG; > 1.7 mmol/l); low high-density lipoprotein cholesterol (HDL; men < 1.0 mmol/l, women < 1.3 mmol/l); raised blood pressure (BP; ≥ 130/85 mmHg or on hypertension treatment); raised fasting glucose (≥ 5.6 mmol/l or on diabetes treatment). Body mass index (BMI) was also calculated. Anthropomorphic measurements were done by a research nurse and laboratory samples were assayed.
3. The Mini International Neuropsychiatric Interview (MINI) [51]: The MINI version 6.0 is a structured diagnostic interview, used to diagnose current and lifetime psychiatric disorders based on DSM-IV and International Classification of Diseases (ICD-10) criteria. The MINI is widely used, takes about 15 min to complete and can be easily administered by trained clinicians and lay interviewers. It has shown good reliability and validity. The MINI was used to determine whether participants met criteria for psychiatric comorbidities and inclusion/exclusion criteria.
4. Clinician-Administered Posttraumatic Stress Disorder Scale for DSM–5 (CAPS-5) [52]: The CAPS is a 30-item structured clinical interview that provides both a PTSD diagnosis and severity score. PTSD diagnostic status was determined based on meeting DSM-5 criteria. Each DSM-5 corresponding item is rated based on the frequency and intensity of the symptoms on a 5-point Likert scale (0 = absent, 1 = mild/subthreshold, 2 = moderate/threshold, 3 = severe/markedly elevated, and 4 = extreme/incapacitating). Symptom severity scores are computed by adding the severity scores for all 20 items (range 0 – 80). Sound psychometric properties for both PTSD diagnostic status and severity scores have been demonstrated [53]. Internal consistency in this sample was good, with the exception of Criterion C (Cronbach’s α, full scale = 0.94).
5. The Life Events Checklist for DSM-5 (LEC-5) [52] and Childhood Trauma Questionnaires (CTQ) [54, 55]: These were used to gather childhood and lifetime trauma-related information. The LEC-5 was developed concurrently with the CAPS-5 to assess lifetime exposure to potentially traumatic events and is administered prior to the CAPS-5. The LEC-5 assesses for exposure to 16 types of potentially traumatic events and an additional item for any other stressful event. The event that participants endorsed as the worst, was used to assess for PTSD symptoms on the CAPS-Events were added up to get a total score. The LEC-5 has demonstrated adequate psychometric properties in both clinical and non-clinical samples [56]. We used the short (28-item) form of the CTQ (CTQ-SF) to assess for maltreatment before the age of 18 years. The CTQ-SF consists of five clinical scales (physical, sexual and emotional abuse, and physical and emotional neglect) of five items each and a minimization/denial scale made up of three items. Each item is rated on a 5-point Likert Scale (1 = never true; 2 = rarely true; 3 = sometimes true; 4 = often true; 5 = very often true). The total severity score (range: 25–125) was used as a dimensional measure of exposure to child abuse and neglect. Adequate psychometric properties have been reported. Internal consistency in this sample was good (Cronbach’s α, full scale = 0.93).
6. The Repeatable Battery for the Assessment of Neuropsychological Status (RBANS), Version A [57]: The RBANS is a brief neurocognitive battery that includes 12 subtests which tap into the following domains: immediate verbal memory and delayed visual and verbal memory, attention, language, and visuospatial skills. It is widely used to assess the neuropsychological function of adults between ages 20 and 89 years, with good psychometric properties reported. The internal consistency in our sample was adequate (Cronbach’s α, full scale = 0.75).
7. Spatial span—from the Wechsler memory scale (WMS-III) [58]: This is a measure of non-verbal working memory/executive functioning. Participants are requested to reproduce increasingly longer sequences of forward and backward taps on blocks. The total score, which is a sum of the forward and backward score was used. It has been reported that WMS-III summary score reliability coefficients range between 0.82 and 0.93 (except the Auditory Recognition Delay Index: 0.74) [59].

Raw scores were used for all measures as local norms were not available.

### Data analysis

Individuals were stratified according to the presence/absence of PTSD and MetS to determine whether PTSD and MetS influenced neurocognition. We compared PTSD patients and controls with Analyses of variance (ANOVAs) for continuous variables (age, income) and Pearson chi-square for categorical variables (sex, employment). We compared those with and without MetS using ANOVA’s. We than performed a between-subjects multivariate analysis of covariance (MANCOVA) on the dependent variables (immediate memory, delayed memory, attention, language, visuospatial performance, working memory and global cognition), controlling for age. Independent variables were PTSD (Patients, Controls) and MetS (Yes, No). Wilks criterion was used to determine significance. and multiple testing.

Based on the above findings we conducted post-hoc exploratory MANCOVAs to determine if individual MetS features influenced cognition, controlling for age. Continuous PTSD severity (i.e., CAPS-5 scores) and MetS feature severity (BMI, WC, glucose, SBP, DBP, TG, and HDL cholesterol levels) were independent variables and cognitive domains were dependent variables. To investigate the impact of each effect on the individual DVs, pair-wise comparisons followed by univariate F-tests were performed.

Data were analyzed using IBM SPSS Statistics, version 29. This study’s design and its analysis were not pre-registered. Two-tailed significance levels were set at p ≤ 0.05, and the trend level was set at p ≤ 0.10.

## Results

### Descriptive data and comparison between patients and trauma-exposed controls on key variables

Patients and TEC were matched with regard to key demographic factors – sex, education, employment and income. Patients were, however, significantly younger than TEC Table 1. With regard to clinical factors, significantly more patients used nicotine in the previous six months, and had exposure to a greater number of trauma types and childhood trauma than TEC Table 2. Patients were also significantly less likely than TEC to meet MetS criteria for glucose.

**Table 1:** Demographic description of PTSD patients and trauma exposed controls

Variable		Patients (N = 236; 49.8%)	TEC (N = 238; 51.2%)	Sig
Sex: N (%)	Male	86 (36.4)	84 (35.3)	χ^2^ (1) = 0.068
	Female	150 (63.6)	154 (64.8)	p = 0.795
Employment: N (%)	Yes	132 (55.9)	136 (57.1)	χ^2^ (1) = 0.071
	No	104 (44.1)	102 (42.9)	p = 0.790
Age (years)		41.8 (11.7)	45.5 (13.4)	F (1472) = 10.372
Mean (SD)				**p < 0.001**
HLOE (years)		10.2 (2.1)	10.3 (2.2)	F (1472) = 0.175
Mean (SD)				p = 0.676
Monthly income: N (%)	Under $180:	120 (53.1)	121 (52.8)	χ^2^ (3) = 1.600 p = 0.659
	$180—$360:	57 (25.2)	59 (25.8)	
	$360 – $720:	34 (15.0)	28 (12.2)	
	More than $720:	15 (6.6)	21 (9.2)	

Numbers may not add up to 100% due to missing data

Significance is denoted in bold text

HLOE: Highest Level of Education; PTSD: Posttraumatic Stress Disorder, TEC: trauma exposed controls

**Table 2:** Clinical, metabolic and neurocognitive description of PTSD patients and trauma exposed controls

Variable		Patients (N = 236; 49.8%)	TEC (N = 238; 51.2%)	Sig
BMI > 30	Yes	105 (44.9)	111 (46.6)	χ^2^(6) = 0.22
N (%)	No	131 (65.1)	127 (53.4)	p = 0.639
HPT criteria met	Yes	129 (57.5)	141 (61.0)	χ^2^(1) = 0.74
N (%)	No	97 (42.5)	90 (39.0)	p = 0.389
Glucose criteria met	Yes	53 (23.6)	89 (38.5)	χ^2^(1) = 12.33
N (%)	No	174 (76.2)	142 (61.5)	**p < 0.001**
HDL criteria met	Yes	93 (39.4)	80 (33.6)	χ^2^(1) = 1.87
N (%)	No	133 (56.4)	149 (63.1)	p = 0.172
TG criteria met	Yes	44 (19.1)	39 (16.4)	χ^2^(1) = 0.45
N (%)	No	182 (76.7)	190 (79.8)	p = 0.501
WC > 90 cm	Yes	106 (45.3)	109 (45.8)	χ^2^(1) = 0.35
N (%)	No	120 (50.4)	119 (50)	p = 0.847
MetS present	Yes	75 (33.2)	83 (30.1)	χ^2^(1) = 0.52
N (%)	No	151 (66.6)	145 (60.9)	p = 0.472
Alcohol use (last 6 months) N (%)	Yes	127 (52.0)	109(47.4)	χ^2^(1) = 1.03
	No	117 (48.0)	121 (52.6)	p = 0.311
Nicotine use (last 6 months) N (%)	Yes	124 (55.4)	100 (44.6)	χ^2^(1) = 5.27
	No	112 (44.8)	138 (55.2)	**p = 0.022**
PTSD severity		35.9 (9.3)	7.6 (8.0)	F (1470) = 1257.51
Mean (SD)				**p < 0.001**
Number of trauma types on LEC		9 (4.5)	6.5 (3.5)	F (1472) = 42.27
Mean (SD)				**p < 0.001**
CTQ total score		58.1 (20.6)	46.8 (17.0)	F (1470) = 48.57
Mean (SD)				**p < 0.001**
Immediate memory		44.2 (7.1)	44.5 (7.6)	F (1452) = 0.25
Mean (SD)				p = 0.62
Visuospatial		32.3 (4.7)	32.1 (5.0)	F (1452) = 0.19
Mean (SD)				p = 0.666
Language		27.5 (4.0)	28.1 (6.3)	F (1452) = 1.91
Mean (SD)				p = 0.167
Attention		45.7 (11.3)	46.2 (13)	F = (1452)0.24
Mean (SD)				p = 0.624
Delayed memory		47.7 (6.7)	47.8 (7.5)	F (1452) = 0.01
Mean (SD)				p = 0.926
Working memory		13.3 (3.7)	13.9 (3.7)	F (1452) = 2.87
Mean (SD)				p = 0.091
Global Cognition (SD)		− 0.09 (0.97)	− 0.05 (1.01)	F (1452) = 2.36 p = 0.125

Numbers may not add up to 100% due to missing data. PTSD Severity: based on the Clinician Administered Posttraumatic Stress Disorder Scale for DSM–5

Significance is denoted in bold text

BMI: Body mass index; CTQ: Childhood Trauma Questionnaire; HPT: Hypertension; HDL: High Density Lipoprotein; LEC: Life Events Checklist; MetS: Metabolic Syndrome (based on harmonised JIS criteria); PTSD: Posttraumatic Stress Disorder; RBANS: Repeatable Battery for the Assessment of Neuropsychological Status; TEC: trauma exposed controls; TG: Triglycerides; WC: Waist Circumference

### Main outcomes

#### Univariate analysis

Relationships between patient status and cognition were first assessed. PTSD patients did not perform worse than TEC on any of the cognitive domains assessed, with the exception of working memory which was lower in patients at the trend level: (F (1452) = 2.87, p = 0.091 (Table 2).

Cognition in individuals with and without MetS was assessed in patients and controls. (Table 3). Attention: F(1450) = 11.40, p < 0.001; language: F(1450) = 3.98, p = 0.047; delayed memory: F(1450) = 4.97, p = 0.026; working memory: F(1450) = 7.63, p = 0.006; and global cognition: F(1449) = 6.62, p = 0.010 were significantly different. Visuospatial Learning trended towards significance: F(1450) = 3.21, p = 0.074; and there were no differences in the immediate memory domain.

**Table 3:** ANOVA comparing cognition in individuals with MetS to those without MetS

	MetS (N = 156)	No MetS (N = 296)	Statistic
Immediate Memory	43.75(7.50)	44.67 (7.22)	F(1450) = 1.62, p = 0.203
Visuospatial Learning	31.62 (4.66)	32.48 (4.94)	F(1450) = 3.21, p = 0.074
Language	28.41 (4.47)	27.37 (5.64)	F(1450) = 3.98, **p = 0.047**
Attention	43.26 (10.90)	47.27 (12.52)	F(1450) = 11.40, **p < 0.001**
Delayed memory	46.71 (7.00)	48.26 (7.07)	F(1450) = 4.97, **p = 0.026**
Working memory	12.89 (3.44)	13.89 (3.78)	F(1450) = 7.63, **p = 0.006**
Global cognition	− 0.19 (0.89)	0.06 (1.02)	F(1449) = 6.62, **p = 0.010**

MetS: Metabolic Syndrome

Significance is denoted in bold text

#### Multivariate analysis

Using the Wilks’ criterion none of the dependant variables were significantly different when controlling for age: Main effect of PTSD: Wilks’ Λ = 0.98, F(6441) = 1.41, p = 0.209, partial η2 = 0.02; Main effect of MetS: Wilks’ Λ = 0.99, F(6441) = 1.02, p < 0.41, partial η2 = 0.01; Interaction effect of PTSD and MetS: Wilks’ Λ = 0.98, F(6441) = 1.54, p = 0.16, partial η2 = 0.02. Age itself, however, was significant: Wilks’ Λ = 0.81, F(6441) = 17.36, p < 0.001, partial η2 = 0.19.

#### Post-hoc analysis

Post-hoc ANCOVA’s were performed to examine the effects of individual MetS features on cognitive domains, controlling for age and PTSD severity (Table 4). Both before and after adjusting for multiple comparisons (adjusted alpha = 0.006), only the effect of BMI was significant: Wilks’ Λ = 0.96, F(6442) = 3.06, p < 0.006, partial η2 = 0.04.

**Table 4:** Effect of Metabolic Syndrome components on cognition, controlling for age

IV	Wilks	F	df	Sig	Eta
MetS factors	0.09	0.69	30,942	0.897	0.02
BMI	0.96	3.06	6442	**0.006**	0.04
SBP	0.99	0.93	6442	0.480	0.02
DBP	0.99	0.81	6442	0.559	0.01
Glucose	0.99	0.90	6443	0.496	0.01
HDL	0.99	1.08	6443	0.374	0.01
TG	0.99	0.38	6443	0.890	0.01
WC	0.99	0.92	6442	0.483	0.01

Significance is denoted in bold text

Eta: Partial Eta Squared; IV: Independent variable; Wilks’: Wilks' Lambda; BMI: Body Mass Index; DBP: Diastolic Blood Pressure; SBP: Systolic Blood Pressure; HDL: High Density Lipoprotein; MetS factors: Number of Metabolic Syndrome Factors; TG: Triglycerides; WC: Waist Circumference

Follow up univariate analysis showed a significant effect for BMI on visuospatial performance: F(1451) = 10.91, p = 0.001, η2 = 0.02; and a trend towards a significant effect for BMI on language: F(1451) = 3.01, p = 0.084, η2 = 0.01.

## Discussion

Our primary aims were to determine whether adult South Africans with PTSD have worse cognitive performance than TEC without PTSD, and whether MetS has an additive effect on the relationship between cognition and PTSD. In addition, we conducted exploratory analysis to determine if individual MetS features, are significantly associated with cognition.

Cognitive difficulties are understood to be involved in the development and maintenance of PTSD [60]. We thus first hypothesized that individuals with PTSD would have poorer cognitive performance than TEC. Despite previous research demonstrating compromised neurocognitive function in PTSD, we did not find this to be the case in unadjusted and adjusted models. PTSD patients did not perform worse than TEC in any of the domains assessed, apart from a trend towards poorer working memory, in the unadjusted model. Similarly, post-hoc analysis of individual MetS features revealed no significant relationships between PTSD severity and cognition.

It may be that due to a high trauma burden and significant socio-economic stressors our sample differs from those in studies that have found differences. Other factors may be that our PTSD control group consisted only of TEC or that we only assessed for current and not lifetime PTSD. The neurocognitive measures we utilized have not all been adapted or validated in our setting and local norms are not available and may therefore not be sensitive enough to detect deficits related to PTSD. Additionally, self-reported cognitive difficulties may not fully align with objective functioning. Indeed, some authors have pointed out that despite subjective complaints of neurocognitive difficulties, these do not always correlate with objective neuropsychological deficits [61]. Age had the most profound associations with cognitive performance. Prior work has documented the effect of demographic variables such as age on cognition [62–64].

Interactions between metabolic syndrome and inflammation may increase risk for cognitive impairment [41, 42, 65]. As such, we hypothesized that individuals with MetS would have poorer neurocognitive performance than those without MetS. We were also not able to prove this hypothesis. Although attention, language delayed memory, working memory and global cognition were significantly different in univariate analysis, they were no longer so in the multivariate model, which controlled for age. Age has been identified as a significant risk factor for both MetS and cognitive impairment [66, 67]. Two-thirds of our sample were women and sex may, therefor, also play a role. A study in a low-income rural setting in China found metabolic risk factors were associated with increased cognitive scores in men but not women [66]. Our study also aligns with a systematic review by Koutsonida and colleagues (2022) who reported that reduced cognition was not consistently associated with the presence of MetS and that most studies reviewed did not report significant relationships between MetS and the cognitive domains investigated [68].

Our third hypothesis was that individuals with both PTSD and MetS would have poorer cognitive performance than those with PTSD or MetS only or those with neither condition. This does not appear to be the case. Earlier reports of poorer cognitiive performance in individuals with both PTSD and MetS are based on veteran populations. Methodological differences between our study and those may thus contribute to the inconsistent findings. Given that the relationships between PTSD, MetS and cognition are not well understood it is important to further examine potential mechanisms underlying these and the effects thereof.

In post-hoc analysis, after adjusting for multiple comparisons and controlling for PTSD severity and age, the only association that remained was that between BMI and visuospatial performance, such that higher BMI was linked with poorer visuospatial performance. Other work has found a relationship between body weight and some aspects of visuospatial performance in both adults and children [69, 70]. The association between obesity and poorer cognition may be affiliated to its role as a marker of vascular and inflammatory damage and that endocrine function and adipocyte leptin secretion may influence the relationship between obesity and brain health [44]. Although PTSD severity was not associated with any of the cognitive domains assessed, the above finding accords with hippocampal system dysfunction, a core feature of PTSD. Individuals with PTSD who demonstrated reduced spatial processing in even neutral/ non-traumatic tasks were found to have reduced hippocampal systems volumes and abnormal white matter integrity to tracts implicated in multisensory integration [34]. Lastly, the presence of more MetS features did not worsen cognition in patients and controls, suggesting that cognitive challenges may be better accounted for by other factors.

Some limitations should be noted: Generalizability may be limited, as participants were recruited using purposive sampling, and were limited to a single ethnicity. This was a cross-sectional study limiting inferences about directionality. Longitudinal studies may help elucidate cause-effect relationships. Whether MetS and its components contribute to cognitive decline or are a consequence of cognitive deficits, resulting in poor decision making such as unhealthy lifestyle choices in those with PTSD, is yet to be determined. Our results may be confounded by the use of TEC only—the inclusion of non-trauma controls in further research may disentangle the effects of the trauma/s themselves and that due to PTSD. Grouping participants according to factors known to be associated with PTSD and MetS i.e., trauma type, number of traumas, gender and age, may reduce heterogeneity and result in different outcomes. Nonetheless, this study adds to our knowledge of the interplay between PTSD, MetS and cognition. To our knowledge, this is the first study to examine this in a community sample or in a LMIC and African setting, using a neuropsychological test battery covering six major cognitive domains- immediate memory, delayed memory, visuospatial performance, attention, language and working memory.

## Conclusion

In conclusion, we found that cognition was not compromised in those with PTSD as compared to TEC, and that MetS and the comorbidity of PTSD and Mets were not associated with cognitive impairments in our sample. Characteristics of this sample may be contributing to these results, given that much research contradicts our findings. BMI, a modifiable risk factor for PTSD and cognitive performance, was however associated with poorer performance in the visuospatial reasoning domain. It is therefore important to interrogate further the potential contribution of MetS to cognitive deficits in those with PTSD, particularly as correction of MetS and its components may improve cognitive function in trauma exposed persons. Well-controlled longitudinal studies are needed to provide insight into the potentially complex association between PTSD, MetS and neurocognition, clarify causal relationships, develop reliable markers and identify potential intervention targets. Studies considering other MetS related components, such as lifestyle factors, and inflammatory markers, may shed further light on this.

## Data Availability

Data are available from the authors upon reasonable request.
